# Computational Exploration of Putative LuxR Solos in Archaea and Their Functional Implications in Quorum Sensing

**DOI:** 10.3389/fmicb.2017.00798

**Published:** 2017-05-03

**Authors:** Akanksha Rajput, Manoj Kumar

**Affiliations:** Bioinformatics Centre, Institute of Microbial Technology, Council of Scientific and Industrial ResearchChandigarh, India

**Keywords:** Archaea, quorum-sensing, LuxR solos, ligand-binding, phylogeny, ecological niche, extremophiles, bioinformatics analyses

## Abstract

LuxR solos are unexplored in Archaea, despite their vital role in the bacterial regulatory network. They assist bacteria in perceiving acyl homoserine lactones (AHLs) and/or non-AHLs signaling molecules for establishing intraspecies, interspecies, and interkingdom communication. In this study, we explored the potential LuxR solos of Archaea from InterPro *v62.0* meta-database employing taxonomic, probable function, distribution, and evolutionary aspects to decipher their role in quorum sensing (QS). Our bioinformatics analyses showed that putative LuxR solos of Archaea shared few conserved domains with bacterial LuxR despite having less similarity within proteins. Functional characterization revealed their ability to bind various AHLs and/or non-AHLs signaling molecules that involve in QS cascades alike bacteria. Further, the phylogenetic study indicates that Archaeal LuxR solos (with less substitution per site) evolved divergently from bacteria and share distant homology along with instances of horizontal gene transfer. Moreover, Archaea possessing putative LuxR solos, exhibit the correlation between taxonomy and ecological niche despite being the inhabitant of diverse habitats like halophilic, thermophilic, barophilic, methanogenic, and chemolithotrophic. Therefore, this study would shed light in deciphering the role of the putative LuxR solos of Archaea to adapt varied habitats *via* multilevel communication with other organisms using QS.

## Introduction

Quorum sensing (QS) is a specialized behavior of microorganisms to coordinate their activities *via cell-to-cell* communication (Miller and Bassler, 2001; Rutherford and Bassler, 2012). It is driven by various species-specific QS signaling molecules (QSSMs) like acylated homoserine lactones (AHLs), QS peptides (QSPs), autoinducer-2 (AI-2), diketopiperazines (DKPs), autoinducer-3 (AI-3), etc. (Rajput et al., 2015, 2016). During the process, QSSMs are synthesized and secreted out from the cells, which further sensed by it or other cells to continue the cascade (Parsek and Greenberg, 2000; Kim et al., 2005). These QSSMs help microbial world to establish diverse vital processes propelled by QS like biofilm formation, secretion of various virulence factors, sporulation, motility, bioluminescence, and many more (Nealson et al., 1970; Henrichsen, 1972; Costerton et al., 1978; Rumbaugh et al., 1999; Yarwood and Schlievert, 2003; Parsek and Greenberg, 2005; Li et al., 2011; Perez-Velazquez et al., 2016).

Acylated homoserine lactones are characterized as the major signaling language for interaction among Gram-negative bacteria. It is processed by various homologs LuxI/LuxR type QS system in bacteria (Miller and Bassler, 2001). Two important proteins, LuxI and LuxR, control the expression of luciferase operon (*luxICDABE*), and thereof are the key regulators of QS circuit. LuxI homolog protein is AHL synthase that catalyzes the reaction between *S*-adenosyl methionine (SAM) and an acyl carrier protein (ACP) to produce AHL molecules (Rutherford and Bassler, 2012). While, LuxR-like proteins activates the transcription of the target DNA by binding to its cognate AHL molecule (Schauder and Bassler, 2001). Moreover, a LuxR homolog protein comprised of two domains, i.e., N-terminal region (response regulatory domain) that binds to its specific autoinducer and C-terminal region with Helix-Turn-Helix (HTH) motif responsible for binding the DNA and hence modulates the expression of genes (Donaldson et al., 1990; Hanzelka and Greenberg, 1995).

LuxR proteins are categorized as canonical LuxR (possessing cognate LuxI) and LuxR solos (lacks cognate LuxI) (Fuqua, 2006). LuxR solos (or unpaired LuxR or bachelor LuxR or orphan LuxR) are proved to sense both AHL and non-AHL molecules and hence termed as AHL or non-AHL binders (Eberhard et al., 1981; Subramoni and Venturi, 2009; Hudaiberdiev et al., 2015). For example, LuxR of *Vibrio fischeri* (Eberhard et al., 1981), TraR of *Agrobacterium tumefaciens* (Zhang et al., 2002), LasR, RhlR, and QscR of *Pseudomonas aeruginosa* (Passador et al., 1993; Pearson et al., 1995; Chugani and Greenberg, 2014), etc. belonged to AHL binders. Whereas PauR from *Photorhabdus asymbiotica* senses dialkylresorcinols (DARs), PluR of *Photorhabdus luminescens* recognizes α-pyrones (Brameyer and Heermann, 2015), PqsR regulator of *P. aeruginosa* binds to 4-hydroxy-2-alkylquinolines (HAQs) (Bala et al., 2013), etc.

LuxI/LuxR based mechanism for QS is extensively explored in Gram-negative bacteria both experimentally and evolutionarily. Various studies regarding the phylogenetic distribution of LuxI/LuxR in alpha, beta, and gamma classes of Proteobacteria (Gram-negative bacteria) was accomplished (Gray and Garey, 2001; Lerat and Moran, 2004; Nasuno et al., 2012; Christensen et al., 2014). Moreover, small subgroups of Gram-negative bacteria like Vibrionaceae (Rasmussen et al., 2014), Roseobacteriacea (Cude and Buchan, 2013), Halomonadaceae (Tahrioui et al., 2013), Aeromonas (Jangid et al., 2007), etc. were also surveyed. Additionally, the autoinducer-binding domain of LuxR solos was analyzed in bacteria on the basis of their distribution and conservation (Subramoni et al., 2015). However, in Gram-positive bacteria (Actinobacteria phylum), few phylogenomic studies were done to check LuxR regulators’ phylogenetic and functional diversity (C-terminal, HTH DNA binding) (Santos et al., 2012; Polkade et al., 2016).

Previously, we have developed a database named SigMol, which encompasses information of all QSSMs reported in prokaryotes (Rajput et al., 2016). Interestingly, in the database few species of Archaea was reported to exploit QS phenomenon. For example, Paggi et al. (2003) studied the presence of intraspecies communication in *Natronococcus occultus* through AHLs and showed their correlation with the production of extracellular proteases. Later on, FilI/FilR regulators were known to process carboxy-AHLs in *Methanosaeta harundinacea* strain 6Ac for cell assembly and carbon metabolic flux (Zhang et al., 2012). Moreover, some archaea like *Methanosarcina mazei, Methanothermobacter thermautotrophicus* (Zhang et al., 2012), *Natrialba magadii* (Montgomery et al., 2013), etc. were also proved to perform cross-talk through QS. However, there is a huge gap in the experimental exploration of QS potential among archaea due to difficulties in culturing them.

Archaea are often considered as “*extremophiles*” found in diverse environmental niche like halophilic, acidophilic, thermophilic, psychrophilic, piezophilic, deep-sea, etc. (Chaban et al., 2006; Sorensen and Teske, 2006; Teske, 2012). Although, biofilm formation is also reported in Archaeal species like *Methanosarcina mazei, Methanothermobacter thermautotrophicus* (Orell et al., 2013), *Ferroplasma acidarmanus* (Baker-Austin et al., 2010), *Sulfolobus* spp. (Koerdt et al., 2011), *Halobacterium salinarum* DSM 3754^T^ (Frols et al., 2012), *Ignisphaera aggregans* (Niederberger et al., 2006), *Thermococcus litoralis* DSM 5473^T^ (Rinker and Kelly, 1996), and many more. However, Archaea are exemplified to exhibit biofilm mode of growth mostly *via* syntrophic interaction with bacteria further proved their active involvement in QS cascade (Frols, 2013; Orell et al., 2013; Perras et al., 2014; Pohlschroder and Esquivel, 2015). Therefore, there is a need to explore the fundamental and vital phenomenon of QS in archaeal species, to uncover various aspect of multilevel communication (intraspecies, interspecies, and interkingdom).

Despite, various bioinformatics resources available for QS like Quorumpeps (Wynendaele et al., 2013), QSPpred (Rajput et al., 2015), SigMol (Rajput et al., 2016), etc., the attempts to explore QS mechanism computationally and evolutionarily in Archaea is lacking. To best of our knowledge, this is the first study that focused on investigating QS in archaea kingdom through multidimensional perspectives. We performed stepwise analyses to unveil the QS potential of LuxR solos in Archaea *via* their distribution, similarity with bacteria, functional characterization followed by correlation between taxonomy and ecological niche.

## Materials and Methods

### Data Collection

For performing bioinformatics survey, all the protein sequences containing LuxI/LuxR domain were extracted from InterPro *v62.0.* It is a meta-database that comprehends sequences from diverse repositories namely Pfam, PROSITE, PANTHER, PRINTS, ProDom, Gene3D, PIRSF, SUPERFAMILY, TIGRFAMs, etc (Hunter et al., 2009). InterPro was searched for the sequences containing LuxI “IPR001690 (Autoinducer synthase)” and LuxR “IPR000792 (Transcriptional regulator, C-terminus), IPR005143 [Autoinducer binding domain (ABD)]” in archaea kingdom as done previously for bacteria by Subramoni et al. (2015).

Amongst all the three domains, only LuxR (C-terminus DNA binding, IPR000792) domain is reported in 110 archaeal proteins (Supplementary Table S1). However, we classified LuxR proteins as solos due to the absence of cognate LuxI domain with them. Furthermore, we used 110 LuxR containing sequences in all the analyses to unveil the functionality of QS in Archaea.

### Multiple Sequence Alignment

Alignment of LuxR archaeal sequences was done with respect to TraR of *Agrobacterium tumefaciens*, to observe the presence of functionally conserved key residues among them (W57, Y61, D70, P71, W85, G113, E178, L182, and G188) (Zhang et al., 2002). The TraR of *A. tumefaciens* was previously used as the reference during MSA for aligning bacterial LuxR sequences (Subramoni et al., 2015). MSA was performed using MAFFT *v7.0*, which employs variants of fast Fourier transform method for identifying homologs regions and alignment (Katoh et al., 2002). Further, the aligned sequences were visualized using MSAReveal.org^1^ software. It disclosed the uniqueness in aligned sequences by showing the statistics for length, % identity, gaps, and consensus.

### Domain Analysis

Domain analysis was done to check the possibility of the conserved portions of protein that can exist, evolve, and function independently from the rest protein chain. LuxR containing proteins was scanned for the presence of all the possible domains (universal) by employing two different strategies. Firstly, the domains among LuxR proteins that are reported in InterPro database were extracted. Secondly, NCBI-Conserved Domain Database (CDD) (Marchler-Bauer et al., 2015) is used for CD-search and only those domains are enlisted that comes out as “*specific hit*.” The outcome of domains by employing both strategies (InterPro and NCBI-CDD) was further explained in two ways, i.e., occurrence of unique domains reported in all proteins and frequency of domain combination. Moreover, pictorial depiction of all domains in 110 LuxR proteins was constructed using Domain Draw tool (Fink and Hamilton, 2007).

### Motif

Motif analysis was done to fetch the structural characteristic (or super secondary) in the protein. Firstly, we extracted the motifs from Archaea LuxR sequences and then scanned them with Gram-negative bacteria to examine the extent of their similarity. Motif discovery and scanning were done using Multiple Em for Motif Elicitation (MEME) and Motif alignment and search tool (MAST) *v4.11.2* software (Bailey et al., 2015), respectively. MEME is used to identify novel and ungapped motifs from the input sequences. Consequently, MAST scans and sorts the sequences by the best-combined match to all extracted motifs by MEME.

### Gene Ontology Annotation

Gene Ontology (GO) annotation enables the assignment of protein functions computationally. GO consortium constructed three structurally controlled vocabularies (ontologies) to portray the gene products linked with biological process, molecular function, and cellular components in species independent mode. GO annotation of LuxR containing archaeal proteins were done using GOA database to find the biological, molecular, and cellular function of the sequences (Ashburner et al., 2000). Molecular function annotation used to describe activities of sequence occurring at the molecular level. The Cellular function provides the information regarding the component of cell where the sequences are active. Moreover, the biological process determines a series of events being driven by some organized assemblies of molecular functions.

In the present study, we focused on highlighting the preference of all the assigned GO (*n*) functions among Archaeal proteins among three different domains of GO. Therefore, combinatorial mathematics based approach was employed. Firstly, we determined a maximum number of GO function combinations (1 to *m*) that can be assigned. Secondly, we extracted a number of proteins involved in all the combinations (1 to *m*) of GO functions. This combinatorial mathematics based approach resulted in the range (maximum to minimum) of proteins that possess common functions. Thirdly, the visualization were done using UpsetR (Lex et al., 2014) package in R. In this study, we gave all the possible information of GO annotation assigned functions like proteins involved in individual function and in all possible intersection sets (number of proteins involves in 2, 3, 4, functions etc.). For example, if all the 110 proteins reported to be involved in five GO functions (e.g., A, B, C, D, and E), then total combinations comes out from the formula given below:

$$Total\;combinations\;=\;\;2^{n}-\;1$$

where, *n* is total number of functions assigned. So, in the case of five GO functions assigned “*Total combinations*” resulted in 31 (= 2^5^ – 1). Further, to know individual patterns (GO function) per combination. Following formula is used:

$$Patterns\;per\;combination\;={\;}_{n}C^{k}$$

where, *n* is total number of functions assigned and *k* is number of combination of which we need to fetch out the patterns [*single* (A, B, C, D), *double* (AB, BC, CD), *triple* (ABC, BCD), etc.]. Therefore, to know the “*patterns per combination*” in which 31 “*total combinations*” are involved of five different GO functions:

$$\begin{array}{c} \begin{array}{c} Patterns\;per\;1\;combination\;\left(single\right)\;=\;5\;(={\;}_{5}C^{1})\;\\ Patterns\;per\;2\;combination\;\left(double\right)\;=\;10\;(={\;}_{5}C^{2})\;\\ Patterns\;per\;3\;combination\;\left(triple\right)\;=\;\;10\;(={\;}_{5}C^{3})\;\\ Patterns\;per\;4\;combination\;\left(quadruple\right)\;=\;\;5\;(={\;}_{5}C^{4}) \end{array}\\ Patterns\;per\;5\;combination\;\left(quintuple\right)\;=\;\;1\;(={\;}_{5}C^{5})\; \end{array}$$

All the combinations and their respective patterns are described in a user-friendly manner by plots in result section of the manuscript.

### Ligand Binding Prediction

Prediction of ligand binding was accomplished to observe the potential of LuxR proteins to involve in QS phenomenon. The ligands were identified using COACH software available in I-TASSER package (Yang et al., 2013). It identifies the ligands using two approaches, i.e., structure-based (TM-SITE) and evolution-based (S-SITES) ligand-binding sites prediction.

### Clustering

Grouping of the LuxR containing protein of Archaea was done to identify the closely related members among all LuxR regulators. All the sequences of archaea were clustered by CLANS (CLuster ANalysis of Sequences) (Frickey and Lupas, 2004). It is a Java-based application, which performs the clustering using network-based approach (unaligned sequences) by employing *all-against-all* BLAST searches and the pairwise attraction values were calculated based on high scoring segment pair *p*-values.

### Phylogenetic Analyses

Phylogenetic analyses were executed to observe the evolutionary relationship of LuxR containing protein of Archaea with other families of LuxR and respective BLAST hits. It was done using Molecular Evolutionary Genetics Analysis (MEGA) 7.0 (Gupta et al., 2016; Kumar et al., 2016). Protein sequences were grouped and aligned using MUSCLE (Edgar, 2004; Hall, 2013) software integrated into same package. Further, the aligned sequences were analyzed phylogenetically employing Maximum Likelihood (ML) method.

The model used for LuxR proteins tree building was WAG (Whelan and Goldman, 2001) with rate variation among sites was formulated with a gamma distribution (shape parameter = 1). Additionally, 16s rRNA tree of 107 sequences (65 archaea and 42 bacteria) was build using Kimura-2-parameter (Kimura, 1980) model. Statistical support for evolutionary analyses was computed by bootstrap in ML (using 1000 pseudo-replicates) along with all the positions with less than 95% site coverage was removed (i.e., fewer than 5% alignment gaps, missing data, and ambiguous bases were allowed at that position).

### Ecological Niche

For correlating the taxonomy and ecological distribution of Archaeal LuxR solos. We extracted the habitat of all Archaea possessing LuxR from various sources like Encyclopedia of life, PubMed, UniProt, JGI genome portal, GenBank, etc. Further, CIRCOS *v0.69* stand-alone software was used to visualize the relationship between taxonomy and habitat of Archaea (Krzywinski et al., 2009).

## Results

### Extent of the Distribution of Putative LuxR Solos in Archaea

**FIGURE 1:**
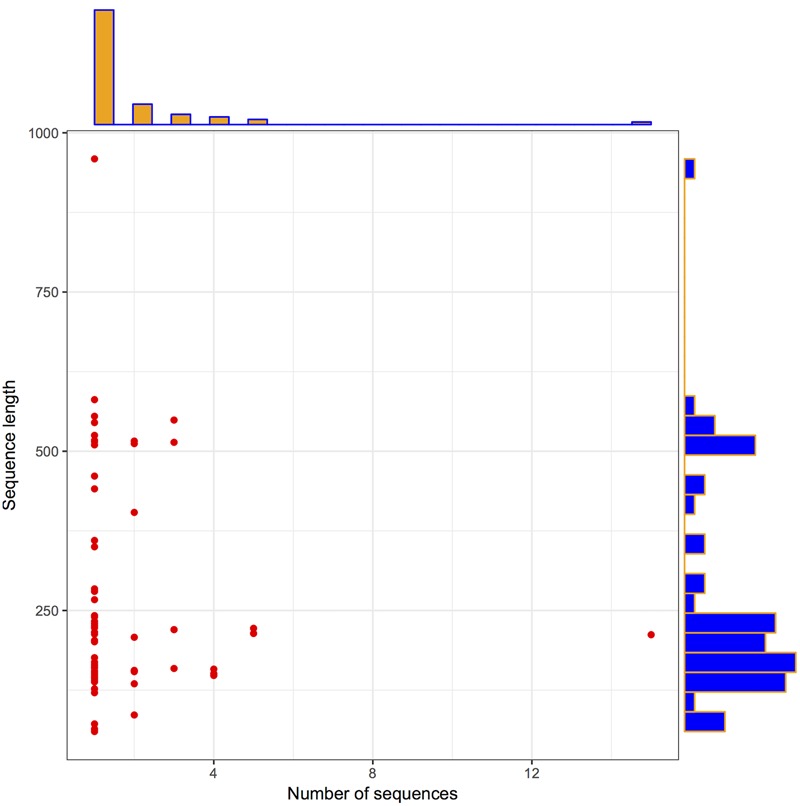
**Archaea LuxR containing sequences statistics.** Scattered plot with the marginal histogram showing a correlation between sequence length and the number of sequences.

To check the distribution of potential LuxR solos in Archaea, InterPro database was explored as described the methodology section. In total, 110 LuxR sequences were obtained from 94 unique Archaea strains. Further, we used 110 LuxR containing sequences for all the analyses, for unveiling their potential to participate in QS. The correlation between their length and frequency was determined to get the brief overview of their distribution. A pictorial summary of archaea sequences (LuxR containing) used in the study depicted as scattered plot with marginal histogram constructed in R for defining sequence length vs. number of sequences in **Figure 1** with the average sequence length of 255 residues.

### Similarity of Putative LuxR Solos of Archaea with Bacteria

**FIGURE 2:**
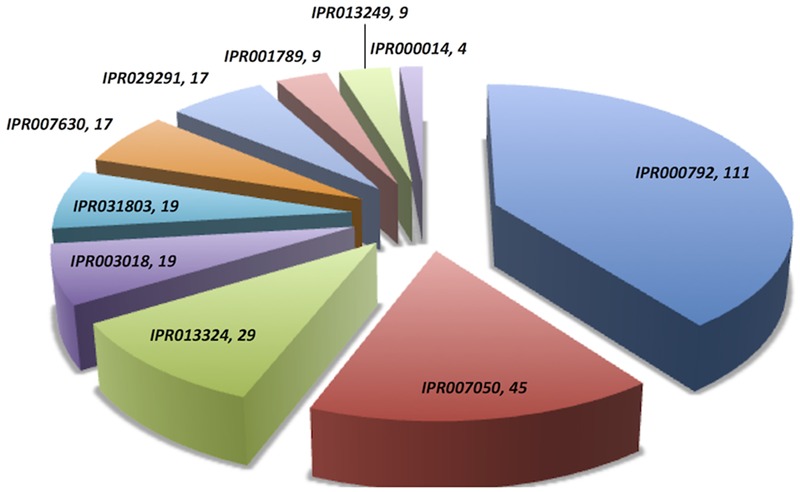
**Distribution of unique domains in 110 LuxR containing archaea sequences extracted InterPro.** [*IPR000792, Transcriptional regulator LuxR, C-terminal domain; IPR007050, Bacterioopsin activator-type, HTH domain; IPR013324, RNA polymerase sigma factor, region 3/4; IPR003018, GAF domain; IPR031803, Bacterioopsin transcriptional activator, GAF and HTH associated domain; IPR007630, RNA polymerase sigma-70 region 4; IPR029291, DNA binding protein Tfx, C-terminal; IPR001789, Signal transduction response regulator, receiver domain; IPR013249, RNA polymerase sigma factor 70, region 4 type 2; IPR000014, PAS domain*].

**FIGURE 3:**
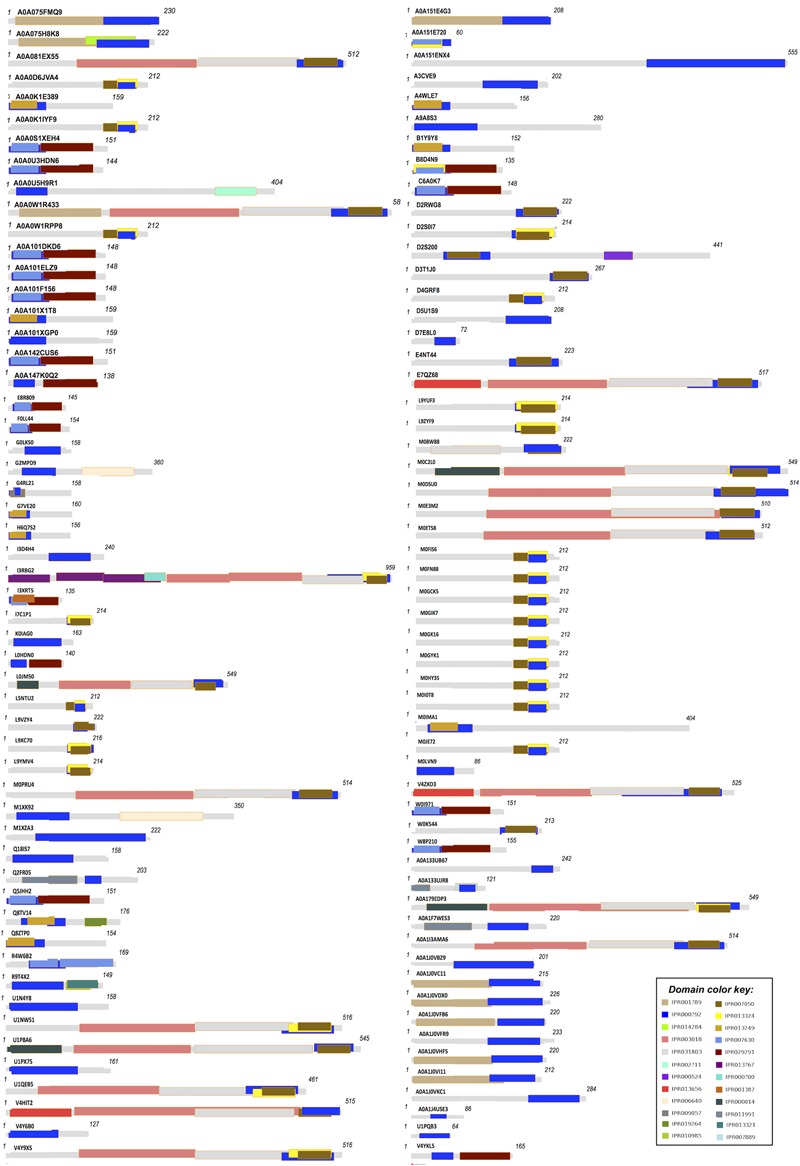
**Domain diagram of 110 LuxR containing Archaeal Proteins**.

Domain extraction was performed to examine the similarity of the independently existing functional unit in Archaea. The presence of all possible domain hits was done using two strategies: (i) InterPro, and (ii) NCBI-CDD database. Moreover, the combinations of all possible domains were also explored in every protein using both the strategies. By using the first strategy, 24 unique domains were reported among 110 LuxR containing archaeal sequences. Top most domain hits belonged to “*Transcriptional regulator LuxR, C-terminal domain*” [IPR000792], and “*Bacterioopsin activator-type, HTH domain*” [IPR007050] in 111 and 45 sites, respectively. Further domains like “*RNA polymerase sigma factor, region 3/4*” [IPR013324]; “*GAF domain*” [IPR003018]; “*Bacterioopsin transcriptional activator, GAF and HTH associated domain*” [IPR031803]; “*RNA polymerase sigma-70 region 4”* [IPR007630]; and “*DNA binding protein Tfx, C-terminal*” [IPR029291] confirmed in 29, 19, 19, 17, and 17 sequences correspondingly. Statistics of top 10 frequently occurring domains are shown in **Figure 2** whereas the list of all the domains along with their occurrence is given in Supplementary Table S2. Moreover, the domain diagram showing the combination of InterPro assigned domains in all LuxR containing Archaeal proteins is provided in **Figure 3**.

On scanning the sequences with NCBI-CDD, 15 different domains were extracted. Few top most domains are HTH_10 (41 hits), GAF_2 (19 hits), BAT (18 hits), HTH_LuxR (16 hits), TFX_C (14 hits), PAS (07 hits), etc. as shown in Supplementary Figure S1. While, exploring the occurrence of domain combination (NCBI-CDD) in LuxR solos with “*specific hits*,” we found “*HTH_10*” in 25 sequences. Whereas other combinations like “*TFX_C*,” “*HTH_LUXR*,” “*BAT* + *HTH_10* + *GAF_2*,” were present in 14, 11, 10, 4, sequences correspondingly. Distribution of top 10 domain combinations extracted by NCBI-CDD given in Supplementary Figure S2. Therefore, it showed that some domains of the putative LuxR solos of Archaea shared similarity with bacteria.

The presence of motifs in putative LuxR solos of Archaea was examined. At *e-value* 1, we extracted 10 motifs using MEME tool that varies in length, sequence coverage from 16 to 50 and 11 to 110, respectively. The detailed information like sequence logo, motif width, regular expression and sequence coverage is provided in Supplementary Table S3. Further, the fetched motifs were searched in Gram-negative bacteria for observing their similarity with them. We found Archaeal LuxR motifs in 14350 out of 73131 Gram-negative bacterial LuxR sequences. The observations suggest that motifs in LuxR solos of Archaea displayed similarity with bacteria LuxR.

Alignment of Archaea LuxR proteins against TraR of *A. tumefaciens* was done to observe the conservation of key residues for ABD and HTH domains. ABD’s residues that make extensive Van der Waals forces with pheromones like W57, Y61, D70, P71, W85, G113 found conserved in 19, 0, 12, 02, 21, and 01 archaeal proteins, respectively. Whereas, HTH binding key residues like E178, L182, G188 present in 25, 48, and 101 LuxR containing proteins. List of proteins possesses key conserved residues are given in Supplementary Table S4. The presence of maximum conserved residues of bacterial LuxR that participate in QS among archaea showed their relatedness with them.

### Functional Characterization of Archaeal LuxR Solos

Gene Ontology analyses carried out for three aspects namely biological process, cellular component, and molecular function.

#### Biological Process

**FIGURE 4:**
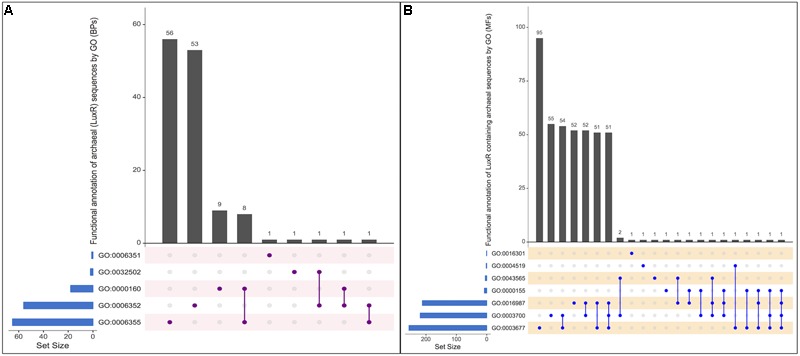
**UpSetR plot showing distribution of Gene Ontology annotating function for LuxR containing proteins of Archaea in (A)** Biological processes, and **(B)** Molecular functions. [*“regulation of transcription, DNA-templated” [GO:0006355]; “DNA-templated transcription, initiation” [GO:0006352]; “phosphorelay signal transduction system” [GO:0000160]; “transcription, DNA-templated” [GO:0006351]; “developmental process” [GO:0032502]; “DNA binding” [GO:0003677], “Endonuclease activity” [GO:0004519]; “Kinase activity” [GO:0016301]; “Sequence-specific DNA binding” [GO:0043565]; “Phosphorelay sensor kinase activity” [GO:0000155]; “Sigma factor activity” [GO:0016987]; “Transcription factor activity, sequence-specific DNA binding” [GO:0003700]*].

From the total biological process, GO annotation functions assigned to LuxR-containing proteins are “*regulation of transcription, DNA-templated*” [GO:0006355], “*DNA-templated transcription, initiation*” [GO:0006352], “*phosphorelay signal transduction system*” [GO:0000160], “*transcription, DNA-templated*” [GO:0006351] and “*developmental process*” [GO:0032502]. Out of total hits number of proteins found to involve exclusively in “GO:0006352,” “GO:0006355,” “GO:0000160,” “GO:0006351,” and “GO:0032502” functions are 56, 53, 09, 01, and 01, respectively. Moreover, few protein annotate to involved in two processes, i.e., “GO:0000160&GO:0006355,” “GO:0006352&GO:0032502,” “GO:0006352&GO:0000160,” and “GO:0006352&GO:0006355.” However, no protein was reported to be involved in more than two biological functions. UpSetR plot showing an overall scenario of biological process assignment is shown in **Figure 4A** and list of archaeal proteins involves in all biological process given in Supplementary Table S5.

#### Cellular Component

Out of total GO annotation for cellular component, scanned archaeal LuxR containing proteins assigned to only three cellular component, i.e., “*intracellular*” [GO:0005622], “*cytosol*” [GO:0005829], and “*plasma membrane*” [GO:0005886]. Ten proteins annotated in the intracellular compartment while 01 protein was found each in cytosol and plasma membrane. UpSetR plot showing the individual and intersecting statistics of proteins are provided in Supplementary Figure S3.

#### Molecular Function

All proteins were assigned to be involved in seven molecular functions like “*DNA binding*” [GO:0003677], “*Endonuclease activity*” [GO:0004519], “*Kinase activity*” [GO:0016301],“*Sequence-specific DNA binding*” [GO:0043565], “*Phosphorelay sensor kinase activity*” [GO:0000155],“*Sigma factor activity*” [GO:0016987], and “*Transcription factor activity, sequence-specific DNA binding*” [GO:0003700]. Among all the functions, maximum proteins involves in “*GO:0003677*” followed by “*GO:0003700*,” “*GO:0016987*,” “*GO:0004519*,” “*GO:0016301*,” “*GO:0000155*,” and “*GO:0043565*” with 95, 55, 52, 01, 01, 01, and 01, respectively. To identify most important molecular functions performed by LuxR containing proteins, we displayed the findings using UpSetR plot (**Figure 4B**) that explains the individual hits correspond to their respective function along with intersection sets of molecular functions in various combinatorial forms. Maximum three molecular functions are preferred by 51 LuxR solos of Archaea, i.e., “*GO:0003677*&*GO:0016987*&*GO:0003700.*” Detailed list of proteins involved in all seven molecular functions given in Supplementary Table S6. Moreover, maximum of these functions are also assigned to bacterial LuxR proteins (data not shown). All the GO annotations confer the active involvement of LuxR containing in signal sensing against environmental cues.

Prediction of potential ligands of Archaeal LuxR proteins was accomplished to characterize their functionality. COACH predictions suggest the presence bacterial QSSMs like AHLs [*N*-(3-oxo-octanoyl)-L-homoserine lactones, *N*-3-oxo-dodecanoyl-L-homoserine lactones, *N*-hexanoyl-L-homoserine lactone, homoserine lactones), peptides, DKPs and α-pyrones (1-deoxy-β-L-tagatopyranose) as ligands in Archaea (shown in **Table 1**). Moreover, various other ligands other than major QSSMs are also reported to be sensed by Archaeal LuxR, e.g., dodecanoyl-CoA, unsaturated fatty acids (1-palmitoyl-2-linoleoyl-sn-glycero-3-phosphocholine) pyruvic acid, amino acids (L-glutamine), metal ion (magnesium (+2), manganese (+2), iron (+3), calcium (+2), etc., c-di-GMP, nucleic acid and many more as provided in Supplementary Table S7. Therefore, the ligands binding potential analysis of the LuxR proteins of Archaea suggested their potential involvement in QS.

**Table 1:** List of ligands predicted to bind LuxR containing proteins of Archaea (40) extracted using COACH software.

UniProt_IDs	Ligands
A0A075FMQ9	Magnesium (+2); trifluoroberyllate (-1); peptide; nucleic acid; c-di-GMP; imido diphosphate
A0A0D6JVA4	*N*-(3-oxo-octanoyl)-L-homoserine lactone; nucleic acid; iron (+3); homoserine lactone; glycolic acid; magnesium (+2); *N*-3-oxo-dodecanoyl-L-homoserine lactone; 1,4-dioxane
A0A0K1IYF9	*N*-(3-oxo-octanoyl)-L-homoserine lactone; nucleic acid; *N*-3-oxo-dodecanoyl-L-homoserine lactone; glycolic acid; magnesium (+2); iron (+3); 1,4-dioxane
A0A0S1XEH4	Nucleic acid; thiamine (+1) diphosphate (-3); *cis*-3,4-dihydrohamacanthin B; L-glutamine; peptide; calcium (+2); xenon; magnesium (+2)
A0A0U3HDN6	6-(2-Fluorobenzyl)-2,4-dimethyl-4,6-dihydro-5h-thieno[2′,3′:4,5]pyrrolo[2,3-d]pyridazin-5-one; 2-phosphoglycolic acid; 3,5-cyclic AMP; sulfate; diphosphate (-2); peptide; calcium (+2); 5-cyclohexyl-1-pentyl-beta-D-maltoside; tetra-MU3-sulfido-tetra iron
A0A0W1RPP8	*N*-(3-oxo-octanoyl)-L-homoserine lactone; nucleic acid; iron (+3); magnesium (+2); glycerol; calcium (+2); GTP
A0A101DKD6	Thiamine (+1) diphosphate (-3); 3,5-cyclic AMP; 1′-deazo-thiamin diphosphate; aldehydo-*N*-acetyl-D-glucosamine; iron (+2); peptide; 2-{4-[(4-amino-2-methylpyrimidin-5-yl)methyl]-5-[(1R)-1,2-dihydroxyethyl]-3-methylthiophen-2-yl}ethyl trihydrogen diphosphate; biselenite ion; Nucleic acid
A0A101X1T8	Nucleic acid; dimethylethylammonium propane sulfonate; chlorophyll A; dodecanoyl-CoA; dequalinium; Cymal-4; pyruvic acid
A0A142CUS6	Oxalate (-2); sulfate; magnesium (+2); 3,5-cyclic AMP; calcium (+2); peptide; chlorophyll A; L-tryptophan
A0A147K0Q2	*N*-cyclohexylcarbamate; L-aspartic acid; hydrogencarbonate; tetra-MU3-sulfido-tetrairon; chlorophyll A; *N,N*,7-trimethylguanosine 5′-(trihydrogen diphosphate); calcium (+2); guanosine-5′-diphosphate; 1-deoxy-beta-L-tagatopyranose
A0A151E4G3	Calcium (+2); trifluoroberyllate (-1); peptide; nucleic acid; c-di-GMP; 3-cyclohexyl-1-propylsulfonic acid; imido diphosphate; NAD zwitterion;
A0A151ENX4	Nucleic acid; peptide; (2E)-3-{3-[3,5-bis(trifluoromethyl)phenyl]-1H-1,2,4-triazol-1-yl}-1-(3,3-difluoroazetidin-1-yl)prop-2-en-1-one; calcium (+2); manganese (+2); sulfate; magnesium (+2)
B1Y9Y8	Nucleic acid; peptide; magnesium (+2); calcium (+2); zinc (+2)
D2RWG8	*N*-3-Oxo-dodecanoyl-L-homoserine lactone; nucleic acid; magnesium (+2); zinc (+2); L-tryptophan
F0LL44	Xenon; iodide; hydrogencarbonate; decyl-beta-D-maltopyranoside; magnesium (+2); *N*-acetylneuraminic acid; 1-deoxy-beta-L-tagatopyranose
K0IAG0	Nucleic acid; chlorophyll A; quinolin-8-ol; peptide; L-aspartic acid; bacteriochlorophyll A
L0HDN0	Nucleic acid; 3,5-cyclic AMP; GDP; oxalate (-2); 1-deoxy-beta-L-tagatopyranose; tetraethylene glycol monooctyl ether; magnesium (+2); calcium (+2); zinc (+2)
L0JM50	2(R),3(E)-Phytochromobilin; biliverdin IX alpha; peptide; calcium (+2); magnesium (+2); zinc (+2)
M0C2L0	Biliverdin IX alpha; peptides; manganese (+2); magnesium (+2); zinc (+2); chlorophyll A; calcium (+2)
M0FIS6	*N*-(3-Oxo-octanal-1-yl)-homoserine lactone; nucleic acid; magnesium (+2); glycolic acid; *N*-3-oxo-dodecanoyl-L-homoserine lactone; homoserine lactone; iron (+3); 1,4-dioxane
M0FN88	*N*-(3-oxo-octanal-1-yl)-homoserine lactone; nucleic acid; magnesium (+2); glycolic acid; *N*-3-oxo-dodecanoyl-L-homoserine lactone; homoserine lactone; iron (+3); 1,4-dioxane
M0GCK5	*N*-(3-Oxo-octanal-1-yl)-homoserine lactone; nucleic acid; magnesium (+2); glycolic acid; *N*-3-oxo-dodecanoyl-L-homoserine lactone; homoserine lactone; iron (+3); 1,4-dioxane
M0GIK7	*N*-(3-Oxo-octanal-1-yl)-homoserine lactone; nucleic acid; magnesium (+2); glycolic acid; *N*-3-oxo-dodecanoyl-L-homoserine lactone; homoserine lactone; iron (+3); 1,4-dioxane
M0GYK1	*N*-(3-Oxo-octanal-1-yl)-homoserine lactone; nucleic acid; magnesium (+2); glycolic acid; *N*-3-oxo-dodecanoyl-L-homoserine lactone; homoserine lactone; iron (+3); 1,4-dioxane
M0HY35	*N*-(3-Oxo-octanal-1-yl)-homoserine lactone; nucleic acid; iron (+3); homoserine lactone; glycolic acid; magnesium (+2); *N*-3-oxo-dodecanoyl-L-homoserine lactone; 1,4-dioxane
M0JE72	*N*-(3-Oxo-octanal-1-yl)-homoserine lactone; nucleic acid; iron (+3); magnesium (+2); glycerol; calcium (+2); GTP
M0LVN9	Magnesium (+2); alpha-D-glucose; peptide; minocycline; 1,4-dioxane; 1-oleoyl-2-palmitoyl-3-alpha-D-galactosyl-*SN*-glycerol; heptaethylene glycol monoethyl ether; iron (+3)
Q5JHH2	Nucleic acid; peptide; calcium (+2); magnesium (+2); sulfate; zinc (+2); L-tryptophan
Q8TV14	Nucleic acid; k-mer; zinc (+2); peptide
V4Y6B0	Nucleic acid; calcium (+2); peptide; magnesium (+2); L-phenylalanine
W0I971	Cyclic AMP; nucleic acid; tetra-MU3-sulfido-tetrairon; magnesium (+2); calcium (+2); peptide
W8P210	Nucleic acid; oxalate (-2); magnesium (+2); sulfate; zinc (+2); peptide; hydrogencarbonate
A0A179EDP3	Biliverdin; zinc (+2); magnesium (+2); manganese (+2); peptide; calcium (+2)
A0A1J0VC11	Manganese (+2); trifluoroberyllate (-1); peptide; xenon; c-di-GMP; imido diphosphate; nucleic acid; 4-cyclopentyl-*N*-[(1S,3R)-5-oxidanyl-2-adamantyl]-2-[[(3S)-oxolan-3-yl]amino]pyrimidine-5-carboxamide
A0A1J0VDX0	Calcium (+2); trifluoroberyllate (-1); peptide; nucleic acid; c-di-GMP; D-tartaric acid; tetrafluoroberyllate (-2); k-mer; xenon
A0A1J0VFB6	Manganese (+2); trifluoroberyllate (-1); peptide; c-di-GMP; magnesium (+2); nucleic acid; D-tartaric acid; k-mer; 3-cyclohexyl-1-propylsulfonic acid; *N*-hexanoyl-L-homoserine lactone
A0A1J0VHF5	Magnesium (+2); trifluoroberyllate (-1); peptide; c-di-GMP; nucleic acid; guanosine-5-RP-alpha-thio-triphspahte; sulfate ion; beryllium trifluoride ion; chloride ion
A0A1J0VI11	Manganese (+2); trifluoroberyllate (-1); peptide; xenon; c-di-GMP; imido diphosphate; nucleic acid; 4-cyclopentyl-*N*-[(1S,3R)-5-oxidanyl-2-adamantyl]-2-[[(3S)-oxolan-3-yl]amino]pyrimidine-5-carboxamide
A0A1J4USE3	Nucleic acid; peptide; heme; magnesium (+2)
U1PQB3	Nucleic acid; L-tryptophan; magnesium (+2); peptide; L-glutamine

### Evolutionary Trend of Putative LuxR in Archaea

**FIGURE 5:**
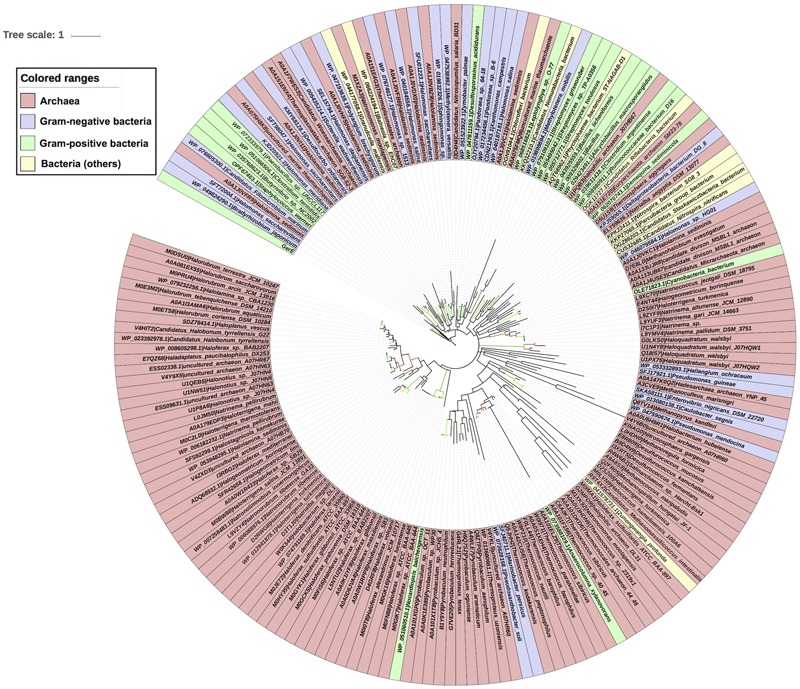
**Phylogenetic analyses of 110 LuxR containing archaeal sequences along with 76 BLAST hits (bacteria and Archaea) using Maximum likelihood tree with bootstrap value of 1000**.

Evolutionary history of archaea LuxR containing sequences was checked by phylogenetic analyses. ML tree for LuxR containing sequence was reconstructed (**Figure 5**) for 110 archaea species along with their respective bacterial or archaeal BLAST hits. The placement of various species in same branch with high bootstrap values explains their high relatedness.

All sequences grouped into two major clades one of archaeal origin and another of bacterial. Archaeal clade was further divided into three sub-clades namely halophilic, methanogenic and thermophilic with high bootstrap values corresponding to their ecological niche.

Each sub-clade further grouped according to species, e.g., halophilic includes *Haloferax* spp., *Natronococcus* spp., *Halogeometricum borinquense, Haloterrigena* spp., *Natrinema* spp., *Natronorubrum tibetense* GA33, *Natronomonas* spp., *Halorubrum* spp., *Haladaptatus paucihalophilus* DX253, etc.; methanogenic consists of *Methanohalobium evestigatum, Methanoculleus marisnigri, Methanospirillum hungatei* JF-1, *Methanococcus maripaludis, Candidatus Methanomassiliicoccus intestinalis* Issoire-Mx1, etc.; while thermophilic incorporates *Pyrobaculum* spp., *Desulfurococcus* spp., *Thermococcus* spp., *Thermococcales* spp., *Thermoproteus* spp., etc.

There are some instances for the presence of members of different ecological niche in another clade with the exception of halophilic sub-clade that contains only halophiles. Whereas methanogenic sub-clade harbors *Haloquadratum walsbyi, Halococcus hamelinensis* 100A6, *Haloferax sulfurifontis* that are halophiles; and thermophilic sub-clade reported to have members of halophiles, ammonia oxidizing archaea (AOA), methanogens and mesophiles like *Salinarchaeum* sp., *Nitrososphaera gargensis, Methanoregula formicica*, and *Euryarchaeota archaeon* SM23-78.

Locations of four species that contain more than one LuxR containing proteins are interesting in phylogenetic tree. As, multiple LuxR copies (*via* gene duplication event) of three species like *Haloterrigena turkmenica* VKM B-1734, *Natronomonas moolapensis* CSW8.8.11, and *Halonotius* sp. J07HQW1 was found at distant places within their respective group halophiles. Moreover, one copy of *Haloferax sulfurifontis* ATCC BAA-897isdistantly placed with *Methanococcus maripaludis* in the phylogenetic tree.

Second major clade is of bacterial species that contains 14 LuxR protein of archaea with namely *uncultured marine thaumarchaeote* AD1000, *Halolamina sediminis, Thermoplasmatales archaeon* SG8-52-4, *Thermosphaera aggregans, Candidatus Nitrosopumilus salaria* BD31, *uncultured marine thaumarchaeote* KM3 and *Thermoplasmatales archaeon* SG8-52-3 and *Candidatus Woesearchaeota archaeon*. Out of them, majority Archaea species branched with Gram-negative bacteria as compared to Gram-positive bacteria. Likewise, 21 bacterial LuxR containing sequences also found in Archaea clade, e.g., *Nocardiopsis baichengensis, Pseudoxanthobacter soli, Marinobacter persicus, Anaerocolumna xylanovorans, Zunongwangia profunda, Pseudomonas mendocina, Caulobacter segnis, Enterovibrio nigricans, Pseudomonas guineae, Haliangium ochraceum, Cyanobacteria bacterium, Halomonas* sp. HG01, *Candidatus Riflebacteria bacterium, Candidatus Nitrospira nitrificans, Candidatus Staskawiczbacteria bacterium, Nitrospira bacterium* SG8 3, *Parcubacteria group bacterium, Deltaproteobacteria bacterium* DG 8, *Demequina sediminicola, Clostridium argentinense*, and *Ruminococcaceae bacterium* D16. However, among them maximum belonged to Gram-negative bacteria group. Although, the grouping of LuxR containing sequences from one ecological niche or kingdom indicates the instances of horizontal gene transfer (HGT) when compared with 16s rRNA gene tree. Moreover, an overall evolutionary analysis revealed that LuxR containing sequences of archaeal origin having low substitution per site as that of bacterial sequences.

**FIGURE 6:**
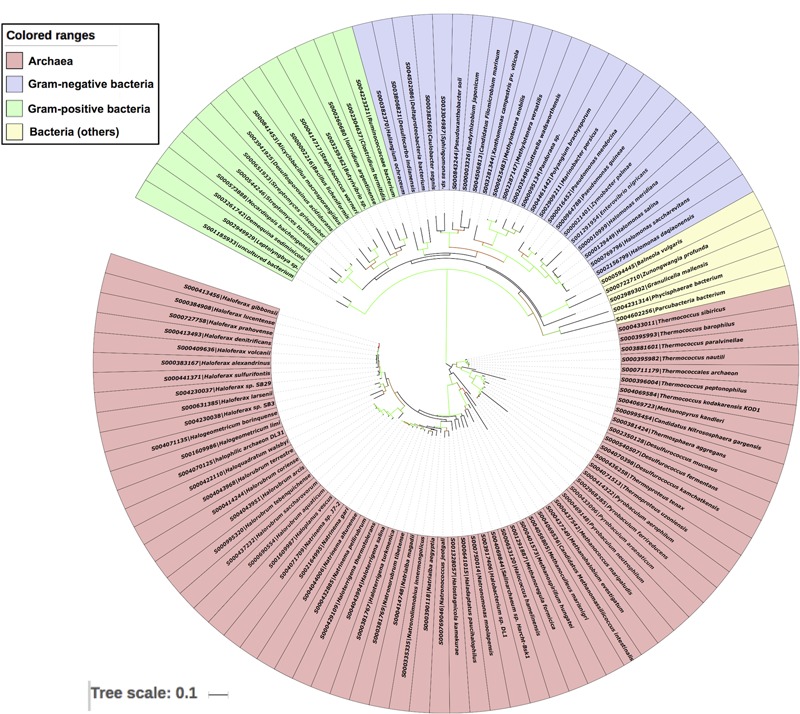
**Phylogenetic analyses of 16s rRNA sequences of 65 archaea along with 42 bacteria using Maximum likelihood tree with bootstrap value of 1000**.

To further validate the LuxR based archaeal phylogeny, we have constructed ML (**Figure 6**) tree of 16s rRNA sequences. The 16s rRNA gene tree showed that all archaea species clustered together and bacterial ones in different clades. Among archaea all members of same species placed together according to their ecological niche with high relatedness among themselves. For example all halophilic archaeal species like *Haloferax* spp., *Halorubrum* spp., *Halogeometricum borinquense, Haloquadratum walsbyi, Haladaptatus paucihalophilus* DX253, *Haloterrigena* spp., *Natrinema* spp., *Natronococcus* spp., *Haloterrigena* spp., *Natronomonas* spp., *Haladaptatus paucihalophilus* DX253, etc. Methanogenic archaea species include *Methanococcus maripaludis, Methanoculleus marisnigri, Methanohalobium evestigatum, Methanopyrus kandleri, Methanoregula formicica, Methanospirillum hungatei* JF-1, and *Candidatus Methanomassiliicoccus intestinalis* Issoire-Mx1. Whereas, thermophilic sub-clade includes species like *Thermococcus* spp., *Desulfurococcus* spp., *Pyrobaculum* spp., *Thermoproteus tenax*, etc. Moreover bacterial clade show diverge branching pattern supported by very high bootstrap support. The analysis showed that bacteria are remote homologs of Archaeal LuxR sequences due to low similarity with them, along with some instances of HGT and gene duplications.

### Distribution of LuxR in Diverse Ecological Niche

**FIGURE 7:**
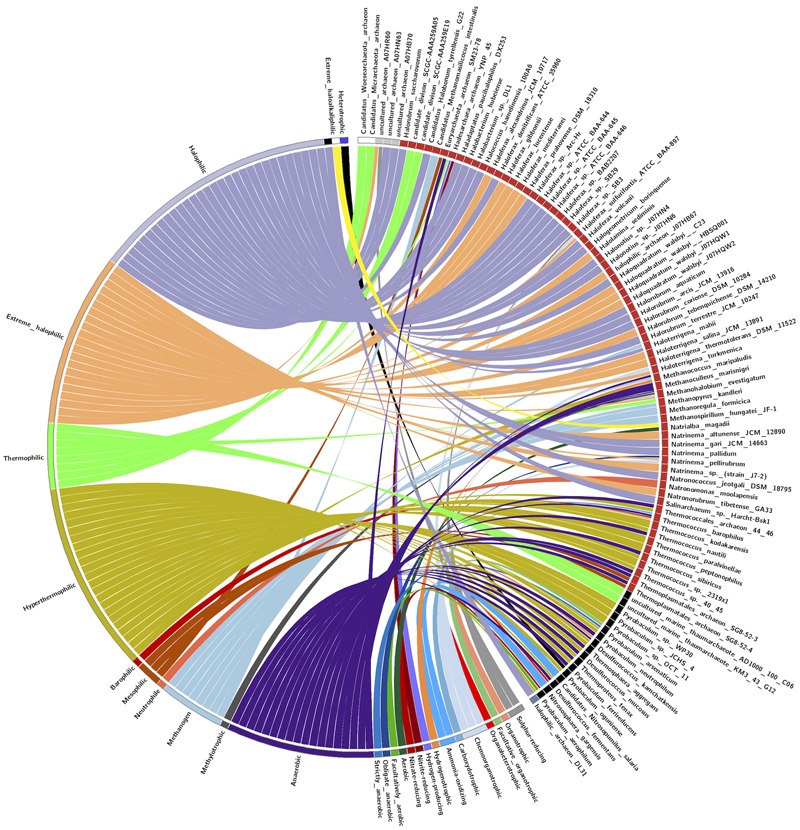
**CIRCOS plot for representing the relationship between taxonomy and ecological niche.** Circle in divided in two parts, rightmost showing 94 unique Archaeal strains and leftmost showing 27 unique habitats. Color of the right arc depicting 05 different groups (white, *DPANN*; gray, *environmental samples*; red, *Euryarchaeota*; black, *TACK*; light blue, *unclassified Archaea*) and left arch and the rays (links) are divided in 27 different colors (gray, *Sulfur-reducing*; light red, *Organotrophic*; green, *Facultative organotrophic*; pale red, *Organoheterotrophic*; very light blue, *Chemoorganotrophic*; blue, *Carboxydotrophic*; pale blue, *Ammonia-oxidizing*; orange, *Hydrogenotrophic*; pale purple, *Hydrogen-producing*; very very dark red, *Nitrite-reducing*; very very dark pale red, *Nitrate-reducing*; very very dark green, *Aerobic*; very very dark pale green, *Facultatively aerobic*; very very dark blue, *Obligate anaerobic*; very very dark pale blue, *Strictly anaerobic*; very very dark purple, *Anaerobic*; very very dark pale purple, *Heterotrophic*; very light dark grey, *Methylotrophic*; light blue, *Methanogen*; red, *Neutrophile*; very very dark pale orange, *Mesophilic*; very dark pale red, *Barophilic*; very very dark yellow, *Hyperthermophilic*; light pale green, *Thermophilic*; light orange, *Extreme halophilic*; light purple, *Halophilic*; black, *Extreme haloalkaliphilic*). Links of 27 colors showing the starting from habitat arc and ending in archaea arc showing their correlation.

The pattern of the distribution of LuxR containing protein in Archaea was examined according to their taxonomy and ecological niche. One hundred and ten LuxR proteins are from 94 unique archaeal species and scattered in 05 different phylum. Maximum archaea belonged to Euryarchaeota followed by TACK, DPANN, environmental samples and unclassified group as shown in **Figure 7**. Predominant habitats are halophilic followed by thermophilic, methanogenic, and anaerobic (**Figure 7**). On correlating the taxonomy and niche specific, we found that most members of Euryarchaeota possessing LuxR proteins are from halophiles or extreme halophiles. Whereas TACK group archaea preferred to be in thermophilic or extreme thermophilic habitat. Moreover, clustering analyses also suggest the significant correlation between taxonomy and habitat. Out of 110 LuxR sequences, 88 remain clustered in 15 groups at *p*-value 1*e* - 30 according to their habitat and taxonomy. Among 15 clusters, eight, three, one clusters are exclusively of halophiles, thermophile, and methanogens. However, three clusters possess species from mixed habitat like halophiles, thermophiles, mesophile, e.g., *Halolamina sediminis, Candidatus Nitrosopumilus salaria* BD31, *uncultured marine thaumarchaeote* KM3_43_G12, *Thermoplasmatales archaeon* SG8-52-4, etc. (Supplementary Figure S4 and Table S8).

## Discussion

LuxR solos are diversely distributed transcriptional regulators in bacteria known to play an important role to sense and respond to environmental cues (Venturi and Ahmer, 2015). They are able to sense internal as well as external signals and helps in the adaptation of microbes despite absence of cognate LuxI (Hudaiberdiev et al., 2015). However, they are well established to involve in QS among bacteria (Patankar and Gonzalez, 2009). Although, till date, they are extensively explored in the bacteria kingdom but their role in Archaea is unexplored. Therefore, in the present study, we tried to explore their distribution in Archaea, the similarity with bacterial LuxR, functional characterization, evolutionary trend and ecological relatedness. Subramoni et al. (2015) searched InterPro database to find the putative LuxR solos proteins. These LuxR solos have ABD and DNA binding domain in bacteria. Likewise, Santos et al. (2012) explored LuxI/LuxR in Pfam database to fetch putative proteins and identified the LuxR regulators with HTH transcriptional factors that involved in QS. We have used the similar strategy to searched LuxI/LuxR in InterPro database and recognized 110 LuxR solos in Archaea that lack ABD and possess only DNA binding, HTH domain.

LuxR solos, well known to be involved in QS were fully characterized and established in Gram-negative bacteria followed by Gram-positive bacteria (Subramoni et al., 2015). While searching their presence in archaea, we found that their frequency is uneven among species; varies from single to maximum seven. Multiple copies of LuxR regulators found in different species, e.g., *Haloferax* spp. followed by *Haloquadratum walsbyi* (04), *Halonotius* spp. (03), *Haloterrigena turkmenica* (03), *Pyrobaculum* spp. (08), *Halolamina sediminis* (08), etc. These archaea thrive in the different extreme environment like high salt, high and cold temperature, high pressure, ammonia and sulfur enriched, etc. and drives various biogeochemical cycles like sulfur, nitrogen, and carbon. Most of the sequences are from halophilic (Enache et al., 2007) archaea followed by thermophilic (Jaakkola et al., 2016), piezophilic (Vannier et al., 2011), methanogenic (Borrel et al., 2013), alkaliphilic (Xu et al., 1999), ammonia oxidizing (Mosier et al., 2012), etc. More than 95% archaea are the inhabitant of aquatic (marine and fresh-water) ecosystem and rest belongs to terrestrial one. Oldest archaea with LuxR domain containing protein is isolated from stromatolites (∼3 billion years) and early cretaceous (∼123 million years) halite was *Halococcus hamelinensis* 100A6 (Goh et al., 2006) and *Halobacterium hubeiense* (Jaakkola et al., 2016), respectively.

Our analyses revealed that LuxR solos of Archaea shared similarity with bacteria and able to perceive small molecules. Although, some domains are not exclusive to archaea but also found in bacteria like Transcription regulator (LuxR, HTH), DNA binding domain, Signal receiver, etc. (Santos et al., 2012; Subramoni et al., 2015). Although, LuxR containing archaeal proteins explored in our study contains various type of domains that indicates the relationship of archaea in signal transduction and its response to wide range of environmental modulators as reported in bacteria (Skerker et al., 2005). Moreover, LuxR based QS signaling is different in Gram-negative (single transcription factors) and Gram-positive (two-component system) bacteria (Sturme et al., 2002). Domains repertoire extracted by our study belonged to one-component and two-component system that are found in Archaea and/or bacteria. Interestingly, we extracted domain from putative LuxR solos of Archaea that are involved in two-component system, which is reported to be acquired *via* HGT from bacteria (Koretke et al., 2000; Ulrich et al., 2005). From scanned domains, MerR and HTH_1 are the exclusive key component of one-component system extracted from putative LuxR solos of Archaea (Ulrich et al., 2005). Whereas domains like GerE, PAS, HTH, etc. are involved in both one-component and two-component systems (Taylor and Zhulin, 1999; Galperin et al., 2001). Moreover, we also found archetypal signal input (*small molecules binding*) domains like PAS, GAF, CheY in putative LuxR solos of Archaea (Galperin et al., 2001; Ulrich and Zhulin, 2010).

HTH motif (*RGL[TS]XEE[IV]A[ED]AL[GD][IV]SRSTV[LS]EH*) of GerE domain present at C-terminal of LuxR proteins in bacteria is reported to involve in signal sensing or QS. Moreover, this motif is also reported in HMM logo in Pfam (PF00196) and sequence logo from PROSITE (PS50043) database as LuxR_HTH motif with their implication in QS. This motif (*Motif 1*) is also present in putative LuxR solos of Archaea. However, the majority of the motifs are conserved according to the ecological niche. Interestingly, alignment results showed 10–25% similarity of Archaeal LuxR solos with bacteria, which are almost same as found among bacterial LuxR solos (18–25%) (Subramoni et al., 2015). Furthermore, we also found substitution among invariant amino acids of ABD that displayed the diversity of LuxR solos to sense wide range to autoinducers (AHLs or non-AHLS).

Gene Ontotology annotation studies showed that Archaeal LuxR solos involved in regulation of transcription through autophosphorylation of a histidine kinase and transfer the phosphate moiety to aspartate that further acts as a phosphor donor to response regulator proteins. However, they also possess sigma factor activity that aids them in making sequence-specific contacts with the promoter elements. Further, they are also annotated to be functional intracellularly in the cell as that of bacterial LuxR regulators (Santos et al., 2012). However, GO-based functional assignment showed that Archaeal potential LuxR solos involved in signal sensing mechanism. Furthermore, to examine their role in QS, the ligand-binding prediction was performed. Although, the analysis showed that Archaeal LuxR solos are functionally characterized by the ability to bind AHLs and non-AHLs ligands as bacterial LuxR solos (Subramoni et al., 2015). It was further supported by MSA, which displayed that among 06 conserved key residues ABD, 05 are found conserved in few Archaea LuxR proteins (Supplementary Table S4). The substitution among invariant amino acids indicates their potential to sense a wide range of signaling molecules (AHLs and/or non-AHLs). However, the presence of AHLs as signaling molecules in Archaea was already reported in SigMol database and various other studies (Paggi et al., 2003; Zhang et al., 2012; Rajput et al., 2016). Additionally, the presence of non-AHL ligands like DKPs was also established in the previous study (Tommonaro et al., 2012). However, other non-AHLs ligands like, α-pyrones, dodecanoyl-CoA, pyruvic acid, amino acids, metal ions, etc. showed their similarity with bacterial LuxR solos (Patel et al., 2013; Brameyer et al., 2014; Brameyer and Heermann, 2015, 2016; Venturi and Ahmer, 2015).

Our analysis revealed that bacteria are remote homologs of Archaeal LuxR protein. The phylogenetic analyses of the LuxR solos protein of Archaea and bacteria showed that they both evolved separately with less substitution per site in archaea as compared to bacteria. However, analyses further confirm the presence of few cases for the transfer of LuxR copies between bacteria and Archaea through HGT. Moreover, placement of multiple LuxR solos copies in same Archaea like *Haloterrigena* spp., *Natronomonas* spp., *Halonotius* spp., *Haloferax* spp. both distantly with different microbial strains and with each other indicates that they are acquired through HGT and gene duplication events, therefore, possessing diverse ligand binding properties like the bacterial LuxR solos copies (Subramoni et al., 2015).

Our study is based on exploring the archaea for an imperative and fundamental phenomenon known as QS. All the analyses showed that Archaea LuxR solos could bind to AHLs and non-AHLs ligands and participate in QS. However, experimental details need to confirm the ligand specificity but difficulties in culturing the Archaea led this kingdom under-explored. Therefore, we used computational approach to explore the extent and functionality of Archaea against QS cascade. Varied computational analyses like similarity, functional characterization and evolutionary history showed their involvement in QS through AHLs and/or non-AHLs ligands. Moreover, potential ecological niche of archaea was collated from literature and correlated with the outcome of our analyses for better understanding for the trend of QS being exploited *via* extremophiles. However, the extent of the diversification for QS in archaea is still a question that needs to be further explored. Simultaneously, the evidence reported in the literature for the occurrence of dominant microbial lifestyle, i.e., biofilms in archaea mostly *via* syntropic interaction with bacteria strengthen our findings that these extremophiles have capabilities perform intraspecies, interspecies, and even interkingdom cross-talks and thrive extreme environment through QS.

## Author Contributions

The idea was conceived by MK and also helped in interpretation, analysis, and overall supervision. Data collection and analyses was performed by AR and MK. The manuscript was written by AR and MK.

## Conflict of Interest Statement

The authors declare that the research was conducted in the absence of any commercial or financial relationships that could be construed as a potential conflict of interest.

## Footnotes

1 http://www.bioinformatics.org/msareveal/
