# Supplementary material for: Computational Exploration of Putative LuxR Solos in Archaea and Their Functional Implications in Quorum Sensing
Source: Front Microbiol. 2017 May 3;8:798. doi: 10.3389/fmicb.2017.00798 (PMC5413776; doi:10.3389/fmicb.2017.00798)
Supplement: Supplementary file 1 [file Data_Sheet_1.DOCX]

**Computational exploration of putative LuxR solos in archaea and their functional implications in quorum sensing**

Akanksha Rajput and Manoj Kumar*

Bioinformatics Centre, Institute of Microbial Technology, Council of Scientific and Industrial Research (CSIR), Sector 39A, Chandigarh-160036, India

* To whom correspondence should be addressed. Email: [manojk@imtech.res.in](mailto:manojk@imtech.res.in)

**Supplementary Material**

**Supplementary Figure S1. Distribution of domains in 110 LuxR containing archaea sequences extracted from NCBI-Conserved Domain database (CDD).** CD search with “*specific hit*” is used to determine domains, CsgD (DNA-binding transcriptional regulator), HTH_10 (Helix-Turn-Helix DNA binding domain), GAF_2 (c**G**MP-specific phosphodiesterases, **a**denylyl cyclases and **F**hlA domain), BAT (GAF and HTH_10 associated domain), FhlA (GAF domain [Signal transduction mechanisms]), HTH_LUXR (helix_turn_helix), TFX_C (DNA_binding protein), PAS (**P**er – period circadian protein, **A**rnt – aryl hydrocarbon receptor nuclear translocator protein, **S**im – single-minded protein) domain, Sigma70_r4_2 (Sigma-70) domain, REC (Signal receiver domain), LuxR_C_like (C-terminal DNA-binding domain of LuxR-like proteins), GerE (Bacterial regulatory proteins), HNHc (NHN nucleases), Response_reg (Response regulator receiver domain), Sigma70_r4 (Sigma 70 domain), HNH (HNH endonuclease), HTH_1 (Bacterial regulatory helix-turn-helix protein), MerR (MerR family regulatory protein).

**Supplementary Figure S2. Distribution of domains combination in 95 LuxR containing archaea sequences extracted from NCBI-Conserved Domain database (CDD).** CD search with “specific hit” is used to determine domains, CsgD (DNA-binding transcriptional regulator), HTH_10 (Helix-Turn-Helix DNA binding domain), GAF_2 (c**G**MP-specific phosphodiesterases, **a**denylyl cyclases and **F**hlA domain), BAT (GAF and HTH_10 associated domain), FhlA (GAF domain [Signal transduction mechanisms]), HTH_LUXR (helix_turn_helix), TFX_C (DNA_binding protein), Sigma70_r4_2 (Sigma-70) domain.

**Supplementary Figure S3. Distribution of LuxR containing sequence of archaea annotated as cellular component domain *via* Gene Ontology.**

**Supplementary Figure S4. Clustering analysis using CLANS software for 110 LuxR containing sequences from archea.** 15 different clusters are shown for different sequences. Clustered sequences are represented, as dots while unclustered are not shown. Yellow dots represent sequences with short length and blue means long sequences. Blast sequence similarity is represented as lines shades ranging from light blue (*p-values*<10^-20^) to dark blue (*p-values*<10^-200^).
